# Accurate contact predictions using covariation techniques and machine learning

**DOI:** 10.1002/prot.24863

**Published:** 2015-08-14

**Authors:** Tomasz Kosciolek, David T. Jones

**Affiliations:** ^1^ Department of Computer ScienceBioinformatics Group, University College LondonGower Street, LondonWC1E 6BTUnited Kingdom

**Keywords:** protein structure prediction, CASP, residue–residue contact prediction, *ab initio* prediction, amino acid covariation

## Abstract

Here we present the results of residue–residue contact predictions achieved in CASP11 by the CONSIP2 server, which is based around our MetaPSICOV contact prediction method. On a set of 40 target domains with a median family size of around 40 effective sequences, our server achieved an average top‐*L*/5 long‐range contact precision of 27%. MetaPSICOV method bases on a combination of classical contact prediction features, enhanced with three distinct covariation methods embedded in a two‐stage neural network predictor. Some unique features of our approach are (1) the tuning between the classical and covariation features depending on the depth of the input alignment and (2) a hybrid approach to generate deepest possible multiple‐sequence alignments by combining jackHMMer and HHblits. We discuss the CONSIP2 pipeline, our results and show that where the method underperformed, the major factor was relying on a fixed set of parameters for the initial sequence alignments and not attempting to perform domain splitting as a preprocessing step. Proteins 2016; 84(Suppl 1):145–151. © 2015 The Authors. Proteins: Structure, Function, and Bioinformatics Published by Wiley Periodicals, Inc.

## INTRODUCTION

The emergence of new covariation‐based contact prediction methods has given a lot of hope for rapid progress in solving the protein structure prediction problem by aiding *de novo* predictions with predicted contact information.^1^, ^2^, ^3^, ^4^, ^5^, ^6^, ^7^ However, it is apparent that the requirement for large and diverse alignments needed for reliable contact predictions has limited the applicability of those methods to only a handful of CASP targets, as most cases where covariation‐based contact predictions are applicable end up falling into the template‐based modeling (TBM) category.^5^ Classical contact prediction methods based on support vector machines or neural networks do not require large alignments and can be used even for the most challenging free modeling (FM) targets, but they suffer from low precision, as was shown in the residue–residue contact prediction category for the previous CASP experiment.^8^ On the other hand, it was shown that the addition of even relatively sparse contact information can improve protein structure models.^9^

At the time of CASP10, the best contact prediction methods based on neural networks or other machine learning approaches, had not yet started to incorporate the as then novel statistical covariation based methods. The highest precision for those methods and for methods evaluated in previous CASP experiments hovered around 20% (considering top‐*L*/5 long‐range contacts) for free modeling targets and was somewhat higher if template‐based modeling targets (TBM‐hard) were included in the evaluation dataset.^8^

For CASP11, we developed a new server based around our recently developed MetaPSICOV method, which takes advantage of the observation that covariation based methods derived using different statistical approaches predict significant nonoverlapping sets of contacts^10^ and also utilizes a well‐established “classic” machine learning contact predictor as an additional source of information.^11^ MetaPSICOV combines these sources of information to (1) increase contact prediction precision and (2) enable making reliable contact predictions for sequences with shallow multiple sequence alignments.^12^

In this article, we present a brief overview of the MetaPSICOV method, as implemented in the CONSIP2 server. We discuss our performance in CASP11 and stress strengths and weaknesses of our approach. We also selected an interesting free modeling case and discuss the model we were able to generate using MetaPSICOV contacts. Finally, we highlight our views on the future development of contact prediction methods and on the obstacles they are likely to encounter.

## MATERIALS AND METHODS

### Contact predictions overview

**Figure 1:**
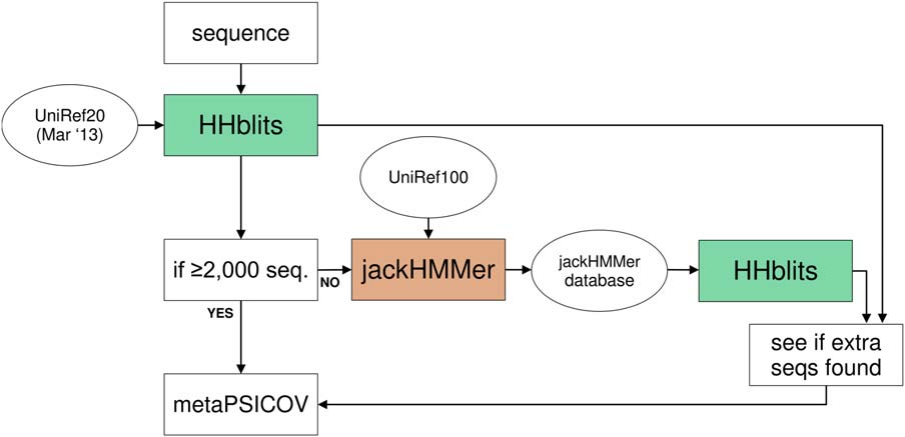
Diagram of sequence alignment pipeline implemented in the CONSIP2 server.

The CONSIP2 server takes the whole submitted target sequence and carries out a hybrid sequence alignment protocol, where we attempt to identify as many homologous sequences as possible using either HHblits^13^ alone, or HHblits and jackHMMer^14^ in tandem (see Fig. 1). The output MSA is then passed on to MetaPSICOV to generate the contact predictions.

The version of MetaPSICOV used in CASP11 combines three covariation based contact prediction methods—PSICOV,^4^ mfDCA,^15^ and GREMLIN.^5^ The covariation and classical contact prediction features are combined using two tandem neural networks. The first stage network generates an initial contact map, which is then passed on to the second stage network, where spurious outliers are removed and gaps in the contact maps are filled. The results are returned for each pair of residues in the query sequence (four residues or more apart) in the form of contact probability estimates.

### Generating sequence alignments

Crucial elements for contact predictions based on amino acid covariation are accurate large and diverse multiple sequence alignments. We developed a workflow to take advantage of as much public sequence data as possible (Fig. 1). By default, the query sequence is used as an input to HHblits with an *E*‐value threshold of 10^−3^, a minimum of 50% coverage and three iterations. Our initial HHblits alignments were generated with the newest available release of the UniRef20 database, which had been last updated in March 2013 and was thus out of date by over a year at the time of CASP11. For comparison, the UniRef100 March 2013 release included 21,824,511 entries, while the May 2014 UniRef100 release (at the start of the CASP11 prediction season) included 36,473,742 entries, that is, it had grown by 67% in the intervening period. In light of this, we attempted to supplement the sequence information for each query sequence. If the HHblits output alignment (A3M file) had <2000 sequences, we constructed an “on the fly” custom database for HHblits using jackHMMer to identify possible homologous sequences in an up‐to‐date release of the UniRef100 database (parameters: *E*‐value = 10, three iterations). Then, the prebuilt custom database, along with the query sequence was used as an input for HHblits. Finally, we compared outputs of both runs—HHblits‐UniRef20 and HHblits‐jackHMMer, and selected the alignment with the most sequences as the input for MetaPSICOV. In this way, HHblits was always used to generate the alignments, but any additional sequences detected in new releases of UniRef100 could be incorporated.

### MetaPSICOV contact predictions

The complete MetaPSICOV approach has already been described elsewhere.^12^ Briefly, MetaPSICOV is a two stage neural network predictor. The first stage uses 672 features in total and generates an initial contact map taking advantage of three covariation‐based contact prediction methods—PSICOV,^4^ mfDCA,^15^ and GREMLIN^5^ (CCMpred^16^ is used instead of GREMLIN in the publicly available implementation of MetaPSICOV; http://bioinf.cs.ucl.ac.uk/MetaPSICOV), mutual information measures^17^ and classical machine learning‐based contact prediction features, such as amino acid profiles, predicted secondary structure and solvent accessibility, along with sequence separation predicted. The second stage takes the output of the first stage (an 11 × 11 window of the full contact map each time) and analyses it to both eliminate outliers and also to fill in the gaps in the contact map. The total number of features used in the second stage is 731.

The covariation methods were selected to represent different approaches to solving the statistical decoupling problem inherent in residue covariation‐based contact predictions (PSICOV—sparse inverse covariance estimation; mfDCA—mean‐field approximation of a maximum entropy inverse Potts model; GREMLIN—pseudolikelihood maximization framework) and, therefore, produce only partially overlapping results. Aside from covariation methods, we used classical neural network‐based contact prediction features (for a full list of features used please refer to MetaPSICOV paper^12^ Table S3). The classical features, whilst having far less predictive power than the covariational features, do play an important role in cases where multiple sequence alignments generated for the query sequence have little depth.

### Effective sequence calculations

Large MSAs can contain many redundant sequences that do not contribute significant contact information. Therefore, like other authors,^7^, ^18^, ^19^ we calculate effective sequence counts (*N*_eff_) rather than raw sequence numbers to account for this redundancy. In our case, we cluster the protein sequences at a 62% identity threshold (which is the same clustering threshold used to compile the standard BLOSUM62 matrix used in BLAST) and then take the number of clusters as the *N*_eff_ value.

### Folding using predicted contacts

To perform *de novo* structure prediction using MetaPSICOV contacts, we used our previously described approach.^20^ In this approach, predicted contacts serve as additional energy terms for FRAGFOLD,^21^, ^22^, ^23^ in addition to the pair‐wise potentials of mean force and solvation. In case of transmembrane (TM) proteins we used FILM3,^24^ instead of FRAGFOLD. FILM3 uses an objective function based on contact predictions alone and distance constraints approximating *Z*‐coordinate values within the lipid membrane.

We used two residue–residue contact potentials—one representing short‐range contacts (E_SR‐RR_, sequence separation < 23) and the other for the long‐range contacts (E_LR‐RR_). The potentials have the form of a square well with exponential decay above the contact distance threshold (8 Å Cβ‐Cβ distance) and are scaled according to the predicted contact probability for each pair of residues with PPV ≥0.5. In the original PSICOV, the contacts were predicted only for the residues where a covariational signal was observed. Therefore, it was suitable to use a single contact‐related function. In case of MetaPSICOV predictions, where contact probabilities for every pair of residues are reported, we found that separating short‐range and long‐range contact potentials yielded better results. Otherwise, the contributions from short‐range contacts (more abundant and easier to predict) outweigh the impact of long‐range contacts what results in lower long‐range contact satisfaction.

## RESULTS

### MetaPSICOV benchmark performance

**Figure 2:**
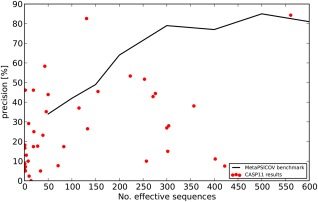
Average MetaPSICOV performance for targets in the small MSA region compared to CASP11 contact prediction results.

We previously benchmarked MetaPSICOV on the PSICOV set of 150 single domain monomeric proteins, which has now served as a benchmark for numerous other contact prediction methods.^4^ In addition, we tested MetaPSICOV's performance on a set of 434 chains from smaller protein families with known experimental structures. Our findings showed that MetaPSICOV performs better than both the consensus of the three individual covariation methods and the neural network (classical contact prediction), regardless of the number of effective sequences in the MSA.^12^ We also found that MetaPSICOV performs on par with any single covariation method, requiring only 200 effective sequences (as opposed to over 600 effective sequences—which was the median of the original PSICOV test set). Considering only top‐*L*/5 long‐range contacts, the prediction accuracy reaches a plateau at around 300 effective sequences giving a mean precision of approx. 80% (Fig. 2).

### Performance in CASP11

In CASP11 there were 40 domains in the contact prediction category (Table 1). Our server achieved an average precision of 27% across all these targets. Using our sequence generation approach (Fig. 1), we were able to generate on average 592 more total sequences using HHblits‐jackHMMer approach, than HHblits‐UniRef20 alone. In all but one cases (T0826‐D1), our method followed the horizontal path of Figure 1 scheme (jackHMMer database for HHblits). The median number of effective sequences calculated using our approach (see Materials and Methods) was 44. The number of effective sequences was calculated from the original alignments generated by the server and truncated to the domain boundaries used in the assessment (provided by the CASP organizers). Therefore, for example, for T0793 which had three of the five domains in the RR analysis, we got a unique value for each of the analyzed domains.

**Table 1:** Summary of CONSIP2 Results

Domain	Length	Top‐*L*/5 LR precision	*N*_eff_
T0761‐D1	88	5.6	1
T0761‐D2	136	8.7	1
T0763‐D1	130	46.2	2
T0767‐D2	180	58.3	43
T0771‐D1	151	10.0	8
T0775‐D2	66	46.2	19
T0775‐D4	61	25.0	20
T0775‐D5	145	0.0	14
T0777‐D1	345	23.2	39
T0781‐D1	200	5.0	2
T0785‐D1	112	18.2	1
T0789‐D1	146	51.7	253
T0789‐D2	126	28.0	304
T0790‐D1	135	44.4	276
T0790‐D2	130	26.9	300
T0791‐D1	156	53.3	223
T0791‐D2	139	42.9	271
T0793‐D1	109	15.0	302
T0793‐D2	45	11.1	402
T0793‐D5	118	38.1	357
T0794‐D2	172	26.5	133
T0799‐D1	141	7.1	2
T0802‐D1	116	13.0	4
T0804‐D2	152	16.7	1
T0806‐D1	256	84.3	561
T0808‐D2	269	35.2	46
T0810‐D1	113	17.4	83
T0814‐D1	137	37.0	115
T0814‐D2	116	82.6	131
T0820‐D1	90	5.6	1
T0824‐D1	108	45.5	155
T0826‐D1	201	7.5	422
T0827‐D2	158	10.0	257
T0831‐D2	244	7.7	71
T0832‐D1	209	2.4	10
T0834‐D1	99	5.0	34
T0834‐D2	92	17.7	28
T0836‐D1	204	43.9	50
T0837‐D1	121	29.2	9
T0855‐D1	115	17.4	19

Contact prediction precision is calculated for top‐*L*/5 LR contacts, where *L* is the length of the protein and LR indicates long‐range contacts (>23 sequence separation).

*N*_eff_—number of effective sequences calculated as described in the Materials and Methods section (see “Effective sequence calculations”).

The results suggest a slightly lower than expected performance, that is 27%, compared to around 30%. Looking at the plot in Figure 2, we can observe some clear outliers, however. We decided to analyze the results of seven of them in more detail (predictions with more than 250 *N*_eff_ and precision below 40%; Table 2). The CONSIP2 server, by default, runs using the whole submitted target sequence, for example, for protein T0793 the server generated a single MSA and predicted contacts for the whole chain, which were then split after submission and assessed independently on a per‐domain basis. Analysing the outliers, we repeated the MetaPSICOV predictions, but constructing the alignments only for the assessed domains in isolation. For six of the seven reanalyzed targets, a drop of between 200 and 400 *N*_eff_ was observed upon realignment and, on average, an increase in their prediction precision due to the removal of drifted or misaligned sequences in the alignment.

**Table 2:** Recalculated Effective Sequence Counts and Precision Values Using Only the Domain Sequence

Domain	Initial		Recalculated	
	Top‐*L*/5 LR precision	*N*_eff_	Top‐*L*/5 LR precision	*N*_eff_
T0789‐D2	28.0	304	36.0	278
T0790‐D2	26.9	300	38.0	258
T0793‐D1	15.0	302	9.5	12
T0793‐D2	11.1	402	25.0	12
T0793‐D5	38.1	357	21.7	61
T0826‐D1	7.5	422	62.5	424
T0827‐D2	10.0	257	20.0	116

Initial results (Columns 2 and 3)—the results submitted by the CONSIP2 server during CASP11 prediction season, obtained using the whole target sequence.

Recalculated results (Columns 4 and 5)—produced by CONSIP2 using only the domain sequence.

The targets generally lost sequences upon realignment. The main reason was because a FM domain (included in the contact assessment) was adjacent to a TBM domain (not assessed here) and thus accumulated more sequences than would otherwise be included in the MSA for the FM domain alone. This would influence MetaPSICOV to overweight the contribution for covariation compared to other features. The other cause of worse than expected performance was anchoring of the alignments toward the more populated easier domain (TBM), resulting in a poor alignment quality for the FM region, but a good alignment for the remainder of the chain.

**Figure 3:**
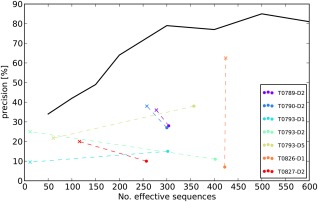
Changes to the outliers upon realigning the domain sequence. The solid line shows reference MetaPSICOV benchmark results. Points represent analyzed outliers: solid circle (o)—initial CONSIP2 server prediction; (x)—result for the realigned domain sequence.

In case of T0793, Domains 3 and 4 were TBM domains and Domains 1, 2 and 5 were FM domains. When the sequences of the FM domains were realigned using only the domain sequences, they lost between 300 and 400 *N*_eff_ compared to the initial result for the whole protein sequence (Fig. 3).

A similar situation could be observed for T0827, T0790 (PDB id: 4L4W), and T0789 (PDB id: 4W4L). T0827‐D2 (FM domain) lost almost 250 sequences upon realignment, gaining 10% precision. T0789‐D2 lost 200 *N*_eff_ gaining 8% precision and T0790‐D2 lost 200 *N*_eff_ but gaining 11% precision.

A different situation was observed for T0826. This target has 2 domains—D2 is a globular TBM domain (not considered here) and D1 is a transmembrane FM domain. When realigned, the number of sequences in D1 did not change substantially (422 vs. 424 effective sequences), but the contact prediction precision significantly increased. In this case, the improvement came about solely because the sequence alignment was no longer anchored to the C‐terminal (TBM) domain.

### Structure predictions using MetaPSICOV contacts

Although we are focussing here on our contact prediction results, the utility of contact predictions can be put in better context by looking at our (group Jones‐UCL) free modeling predictions made using our predicted contact lists. In every FM case, CONSIP2 server predictions and alignments were manually inspected for domain boundaries, alignment errors, or transmembrane targets and rerun, if needed.

**Figure 4:**
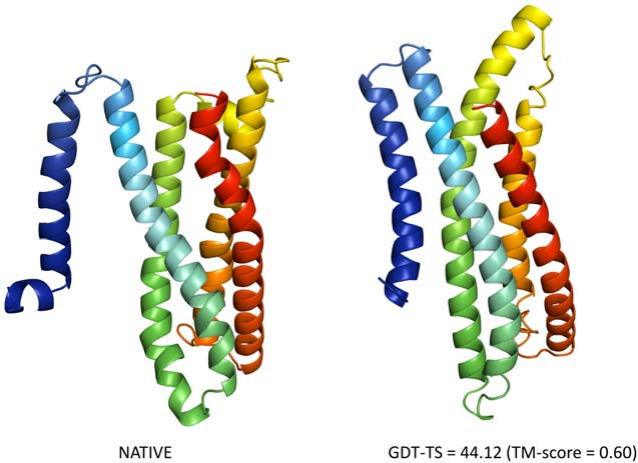
Jones‐UCL FM prediction for target T0836‐D1. A sample free modeling case, where the predicted contacts (*N*_eff_ = 50; top‐*L*/5 LR contact precision = 44%) helped to produce an outstanding model.

One interesting case of a successful usage of predicted residue–residue contacts is that of T0836, which is a 5‐helix transmembrane target, as determined by MEMSAT‐SVM^25^ and confirmed by visual inspection. It allowed us to use our TM protein modeling protocol—FILM3.^24^ For T0836 we were able to find only 50 effective sequences, which would not have been sufficient to allow us to apply the original FILM3 method based on PSICOV alone. Nevertheless, using MetaPSICOV contacts (top‐*L*/5 precision = 44%) we were able to produce a model with TM‐score = 0.60 (Fig. 4). All five of our submitted models ranked in the top seven of the ranking according to GDT‐TS.

### What went right?

We were able to achieve a substantial improvement in the contact prediction precision, with a satisfactory precision at only 200 *N*_eff_, whereas for a single covariation‐based contact prediction method we would have required around 600 *N*_eff_. It is clear that using multiple covariation techniques we are able to improve significantly over classical machine learning contact predictors. Although with each edition of CASP both datasets and methods evolve, it is clear (also from our comparison of network‐only MetaPSICOV^12^) that covariation methods give us an important new source of residue contact information.

More importantly, we were able to further confirm that it is possible to successfully apply predicted residue–reside contacts in structure predictions of large FM targets. In 8 of our (group Jones‐UCL) FM targets (including two transmembrane proteins) we were able to achieve GDT‐TS scores > 40 (TM‐score > 0.5).

### What went wrong?

The analysis of outliers in our predictions clearly suggests that multi‐domain proteins should be processed per‐domain. The current CONSIP2 (and MetaPSICOV) server accepts the whole sequence and does not attempt to split the sequence into domains. This resulted in some subpar predictions. We hope to address this shortcoming before the next CASP experiment.

Another aspect is setting the sequence coverage criteria appropriately. On one hand, a too stringent sequence coverage requirement might result in losing some sequence information for targets where the sequence data is already scarce. On the other hand, a too permissive sequence coverage requirement could result in finding partial matches for some targets and allowing for insufficient coverage in multi‐domain proteins. It is generally easy to spot and correct these issues when looking at the alignments by eye, but difficult to come up with a completely automated system that can deal with the wide variety of scenarios across the whole range of CASP targets.

Our method was also designed to tackle the contact predictions for globular domains as the most general case. In the case of transmembrane domains, building too deep alignments could result in unrelated sequences or drifted domains being included and thus generating false positives in the predicted list of contacts. The notable case here is T0826‐D1, where we included over 400 sequences in the alignment but the contact prediction accuracy was very poor (precision below 10%). Thanks to manual tweaking in the FM category and rerunning the contact predictions on a new alignment, we were able to obtain a satisfactory model and a high contact prediction precision (above 60%).

CONSIP2 was created to achieve the deepest achievable sequence alignments by constructing HHblits databases on the fly. It would, however, be more desirable to have a complete up to date HHblits database. We realize that clustering over 35 million sequences is a serious computational endeavour, particularly for a single research group, but perhaps this is a challenge best handled by a community‐wide distributed computing approach, as sequence alignments are required universally and public sequence data is growing at an ever increasing rate.

## DISCUSSION

In CASP11, we were able to improve the state‐of‐the‐art in residue–residue contact prediction by combining classical contact prediction features with three covariation‐based methods, within a neural network framework. In our approach, we put an emphasis on constructing the largest possible multiple‐sequence alignments. An important aspect of MetaPSICOV is its ability to modulate the relative impact of covariation‐based and classical features depending on the quality and depth of the MSA. If the alignment is shallow, then covariation features are downweighted, whereas for deep and diverse alignments the covariation features are dominant. It is possible that further improvements of the way this is currently handled could result in further increases in prediction accuracy for smaller numbers of sequences, and this is clearly a focus point for future research.

Overall, we were able to achieve a precision of 27% on the CASP11 contact prediction set which had a median of 44 effective sequences (36 after recalculating overestimated domains). Our CASP11 results probably show reasonably well what are the realistic expectations for what contact predictions can contribute to the *de novo* prediction problem for different sizes of MSAs. MetaPSICOV works very well at approximately 200 *N*_eff_ and above, so although we were able to significantly decrease the required number of sequences in an alignment, there is still a gap between what is feasible for sequence family sizes as they currently are, in the majority case at least, and what is needed to exploit these modeling techniques for really challenging cases. It is clear, that with the current methods it is impossible to solve the protein structure prediction problem with the use of contact predictions alone unless we accumulate many more sequences or are better able to deal with alignments with fewer effective sequences.

Improvements in this area could come from two directions. One is the evolution of contact prediction algorithms. On the side of covariation, the major issues of indirect coupling and phylogenetic bias were ameliorated in recent years, but are still unresolved.^10^, ^26^, ^27^ Nevertheless, this progress alone is not going to solve the problem. In case of very small MSAs there is simply not enough covariation information to analyze, no matter what algorithm is applied.

The other issue is the availability of sequence data. We observe an increase in the median Pfam family size,^20^ but it is still unclear how the relative sizes of available sequence and structure data are going to evolve in the coming years.^5^ As we have said before, we do not believe that new sequencing technologies will be able to deal with this problem alone, as for proteins inhabiting particularly unique evolutionary niches, there may only exist very limited numbers of homologous sequences in nature with sufficient sequence diversity to carry out covariation analysis. Time will tell of course.
